# Separating Proactive Conservation from Species Listing Decisions

**DOI:** 10.1007/s00267-022-01713-9

**Published:** 2022-09-13

**Authors:** Adrienne I. Kovach, Amanda E. Cheeseman, Jonathan B. Cohen, Chadwick D. Rittenhouse, Christopher M. Whipps

**Affiliations:** 1 Department of Natural Resources and the Environment, University of New Hampshire, Durham, NH USA; 2 South Dakota State University, Natural Resource Management, Brookings, SD USA; 3 Department of Environmental Biology, State University of New York, College of Environmental Science and Forestry, Syracuse, NY USA; 4 Department of Natural Resources and the Environment, University of Connecticut, Wildlife and Fisheries Conservation Center, Storrs, CT USA

**Keywords:** Cooperative conservation, Endangered Species Act (ESA), New England cottontail, Proactive Conservation, PECE policy, Species status assessment

## Abstract

Proactive Conservation is a paradigm of natural resource management in the United States that encourages voluntary, collaborative efforts to restore species before they need to be protected through government regulations. This paradigm is widely used to conserve at-risk species today, and when used in conjunction with the Policy for Evaluation of Conservation Efforts (PECE), it allows for successful conservation actions to preclude listing of species under the Endangered Species Act (ESA). Despite the popularity of this paradigm, and recent flagship examples of its use (e.g., greater sage grouse, *Centrocercus urophasianus*), critical assessments of the outcomes of Proactive Conservation are lacking from the standpoint of species status and recovery metrics. Here, we provide such an evaluation, using the New England cottontail (*Sylvilagus transitionalis*), heralded as a success of Proactive Conservation efforts in the northeastern United States, as a case study. We review the history and current status of the species, based on the state of the science, in the context of the Conservation Initiative, and the 2015 PECE decision not to the list the species under the ESA. In addition to the impacts of the PECE decision on the New England cottontail conservation specifically, our review also evaluates the benefits and limits of the Proactive Conservation paradigm more broadly, and we make recommendations for its role in relation to ESA implementation for the future of at-risk species management. We find that the status and assurances for recovery under the PECE policy, presented at the time of the New England cottontail listing decision, were overly optimistic, and the status of the species has worsened in subsequent years. We suggest that use of PECE to avoid listing may occur because of the perception of the ESA as a punitive law and a misconception that it is a failure, although very few listed species have gone extinct. Redefining recovery to decouple it from delisting and instead link it to probability of persistence under recommended conservation measures would remove some of the stigma of listing, and it would strengthen the role of Species Status Assessments in endangered species conservation.

## Introduction

Proactive Conservation––often specifically called Cooperative Conservation or Collaborative Conservation––is a paradigm of natural resource management in the United States (U.S.) that emerged in the 1990s and 2000s and is the current model for conserving at-risk species that are candidates or likely future candidates under the Endangered Species Act (ESA) (Duvall et al., 2017). Proactive Conservation refers to voluntary efforts towards species and habitat restoration that take place before the need for legal, governmental protection. Conceptually, Proactive Conservation has its roots in international initiatives such as “participatory development” and “community-based conservation”, which emphasize shared responsibility between governments and local communities in managing natural resources (Conley and Moote, 2001). As such, it is a bottom-up approach that engages diverse stakeholders in collaborative efforts for natural resource management. Based in socioecological systems theory, Proactive Conservation values collaborative decision-making and shared governance and is motivated by the belief that solutions to environmental challenges must be inclusive of societal, political and economic dimensions, along with the ecological (Duvall et al., 2017; Klinger et al., 2007).

The Proactive Conservation paradigm entered federal policy as the U.S. Fish and Wildlife Service (USFWS) and the National Marine Fisheries Service encouraged the development of collaborative conservation plans, funded by challenge grants in the George W. Bush (U.S. president 2000-2008) era, as a means to achieve voluntary conservation efforts based on cooperation and negotiation rather than the regulatory hammer of the ESA (Uchitel, 2005). In 2003, the Pre-existing Conservation Efforts (PECE) Policy formalized the legal requirement for considering pre-listing conservation efforts in the ESA listing decision process (USFWS, 2003a). Specifically, PECE stipulates that listing may be precluded if ongoing conservation efforts are judged to have a high level of certainty in their implementation and effectiveness for promoting the species’ persistence. Since then, several federal policies have been implemented to incentivize collaborative pre-listing conservation efforts, including Candidate Conservation Agreements (CCAs), Candidate Conservation Agreements with Assurances (CCAAs), and Voluntary Prelisting Conservation Actions. Candidate species are those for which the USFWS has determined that listing “is warranted but precluded”, where precluded refers to a lack of resources for the listing process given other species that are a higher priority (Ortiz, 1998). These policies, through the USFWS’s Candidate Conservation Program, are intended to “facilitate cooperative conservation of species that are candidates or likely to become candidates for listing in the near future under the ESA, so that listing is unnecessary.” (USFWS 2018).

Proponents of Proactive Conservation tout benefits of broad engagement of partners with diverse interests and values. This participatory capacity enhances perceptions of legitimacy for stakeholders, minimizes marginalization of groups, facilitates learning among partners, and results in a more integrated approach to governance that may foster resilience of the whole system rather than a fragmented, single species approach (Cosens et al., 2017; Gosnell et al., 2017). Indeed, cooperative conservation of biodiversity can effectively leverage opinions from multiple experts and facilitate the pooling of resources to conduct large-scale management interventions. These collaborations are necessary because 1) the best available science is often lacking or incomplete for imperiled species, and 2) identified management interventions often cross jurisdictions and require multiple partners to succeed (Lauber et al., 2011).

Proactive Conservation also has the advantage of improving participation by stakeholders affected by wildlife management, a perpetual concern with implementation of the ESA. Perception of the ESA’s provisions as punitive against landowners often prevents stakeholders from trusting conservation agreements for listed species. It is important to overcome such barriers, because for the many conservation-reliant species currently listed as threatened or endangered, the factors that led to endangerment are not likely to subside after delisting; consequently, long-term agreements to manage species in perpetuity represent the only chance for recovery to be sustainable (Goble et al., 2012). Protection from the regulatory burden of the ESA is a strong motivating factor in the Proactive Conservation approach, as private landowners are more inclined to participate in endangered species conservation when the approach is cooperative or incentive-based, rather than punitive (Hansen et al., 2018).

Without the provisions of the ESA, however, there are no consequences for failure of collaborative agreements, as defined by species extinction. Therefore, when Proactive Conservation is used to avert species listing, there are no regulatory mechanisms to serve as a backstop against failure or to compel re-evaluation of the process over time. For example, greater sage-grouse conservation (*Centrocercus urophasianus*) has been held up as a model for stakeholder engagement, arriving at consensus on best available science and collaboratively devising goals to meet the values of multiple partners (Allen et al., 2017; Duvall et al., 2017). The effort was widely heralded for precluding listing of the greater sage-grouse under the ESA, although it had been determined that the species status warranted listing prior to the cooperative partnership process (Allen et al., 2017). Individual states enacted conservation programs, including reduction of development-related disturbance in core areas in Wyoming (Smith et al. 2016, Gamo and Beck 2017), consultation requirements and a credit system for mitigating habitat development in Montana (Sime 2016). The population of greater sage-grouse has declined every year since the cooperative partnership process convened, including a 24% decline in Oregon between 2018 and 2019 (Foster, 2019). Range-wide, abundance has decreased by 37% in the last 20 years and 30% of the population is at risk of extirpation in 50 years (Coates et al. 2020). Because the greater sage-grouse is not listed, there is no legal impetus to revisit management until there is a new petition to list the species. In fact, the first new presidential administration after the cooperative plan was enacted lifted its habitat protections, and soon thereafter Congress used federal spending bills to prohibit the USFWS from revisiting the listing process (McGlashen, 2019).

Like the case of the greater sage-grouse, conservation of the New England cottontail (*Sylvilagus transitionalis*) has been hailed as a flagship success of Proactive Conservation. In September 2015, after it spent nine years as a candidate for endangered species status due to an 86% range contraction and population decline, the USFWS declined to list the New England cottontail under the ESA (USFWS, 2006, 2015a). The New England cottontail was touted as a model of Cooperative Conservation, whereby the impetus for the decision drew on an established range-wide federal, state, and private partnership engaged in ongoing restoration activities intended to recover the species (USFWS, 2015a). Five years later, monitoring efforts for the New England cottontail continued to point toward range-wide decline (Rittenhouse and Kovach, 2020), raising concern for the success of ongoing recovery efforts.

Considering the many challenges of ESA implementation and concerns about its regulatory burdens (Buck et al., 2009; Stevens and Conway, 2019), the premise of pre-listing (proactive) conservation efforts is appealing: engage key stakeholders in a shared governance model with socioecological benefits (Madden and McQuinn, 2014) and flagship examples of its success (Lauber et al., 2009). Yet, if Proactive Conservation continues to replace the ESA regulatory process for conserving at-risk species, it is necessary to take a critical look at the outcome of the collaborative efforts from the standpoint of species status and recovery metrics. To our knowledge such critical assessments are lacking to date.

Our goal in this paper is to evaluate the current Proactive Conservation paradigm using the New England cottontail, one of the most widely hailed recent examples, as a case study. We structure the paper into five sections. First, we describe the history of the species status and decline and the rise of the Conservation Initiative. Next, we present the state of the science with respect to ecology and status––we briefly review what was known at the time of the decision not to list and describe what has been learned in the seven years since. We then discuss the remaining threats and uncertainties to population persistence. In the remaining sections, we reflect on the successes and challenges of the conservation initiative and provide suggestions for leveraging what has been learned to forge a more successful path toward species recovery. We conclude the paper with an evaluation of the benefits and limitations of the Proactive Conservation paradigm and make recommendations for its role in relation to ESA implementation for the future of at-risk species management. Our review and evaluation are focused on the PECE and Proactive Conservation paradigms and their implications and are not meant to diminish the substantial efforts and achievements of the dedicated partners involved in the New England Cottontail Conservation Initiative.

## Decline of the New England Cottontail and Rise of the Conservation Initiative

**Fig. 1:**
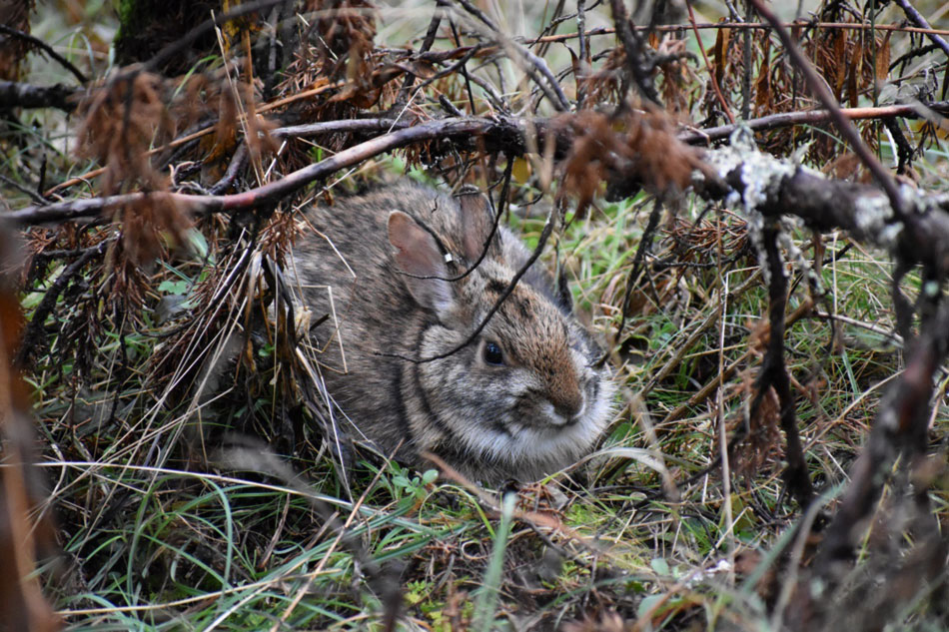
New England cottontail (*Sylvilagus transitionalis*) resting under downed red cedar

**Fig. 2:**
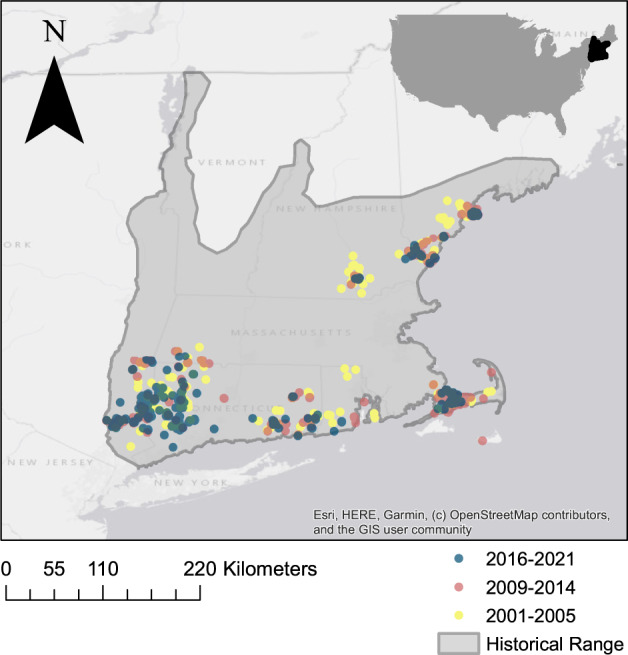
Map showing decline of New England cottontail distribution in the northeastern United States, with historical range (gray shading, adapted from Nelson 1909) and contemporary detections recorded from surveys over the last two decades, including the 2001–2005 range-wide survey of Litvaitis et al. (2006; yellow points), 2009–2014 surveys (red points), and 2016–2021 occupancy surveys (blue points). Previously unpublished data (2009–2014 and 2019–2021) were used with permission of the New England Cottontail Conservation Initiative

The New England cottontail, the only rabbit native to New England and eastern New York, United States, is a shrubland obligate species that exclusively occupies the dense understory vegetation common in early successional and young forest ecosystems, more persistent forested ericaceous and coastal shrublands, and densely vegetated wetlands (Barbour and Litvaitis, 1993; Chapman, 1975; Cheeseman et al., 2018; Litvaitis, 1993; Fig. 1). Shrubland loss and fragmentation have become widespread in the northeastern US as a result of land use change, including wetland conversion, development, agricultural abandonment, and natural succession and reforestation, combined with the loss of natural disturbance regimes responsible for successional shrubland creation (Brooks, 2003; Litvaitis, 2003; Lorimer and White, 2003; Tiner, 1984, 1988). Due to strict habitat specialization, limited dispersal, and patch size requirements, the New England cottontail is one of the most severely impacted of the many shrubland-dependent species experiencing population declines concomitant with the loss of these landcover types in the northeastern US. Restoration of early successional landcover types to augment populations of declining species is a conservation priority in this region, and the New England cottontail, persisting in a small fraction of its historical range (Brubaker et al., 2014; Fenderson et al., 2014; Litvaitis et al., 2006); Fig. 2), has served as a focal species in this effort.

Historically, the New England cottontail was distributed widely throughout all of Connecticut, Massachusetts, and Rhode Island, most of Vermont, New York east of the Hudson River, a large part of New Hampshire south of the White Mountains, and in Maine from Augusta to the Penobscot Bay area (Chapman, 1975; Hall and Kelson, 1959; Litvaitis et al., 2006; Nelson, 1909). This distribution includes the physiogeographic regions of the Coastal Lowlands (New England Coastal Plain), which extends from the Connecticut coast to northeastern, coastal Maine, and an inland upland zone that extends from east of the Hudson River in southern New York and across the inland portions of Connecticut and Massachusetts across to New Hampshire (Foster and Aber, 2004). Origins of the cottontail in these areas are thought to predate written records (Jackson, 1973; Fig. 2). Some reports suggest there may have been a northward expansion of the New England cottontail in the late 1800s and early 1900s, resulting in a distribution in the early 1900s that was expanded relative to historical (precolonial baselines) (Bangs, 1895; Jackson, 1973; Palmer, 1944; William, 1888). Based on these reports, the species’ historical distribution was recently re-defined to exclude the northernmost portion of its current range in southwestern Maine (USFWS, 2015b). This redefined distribution, however, places an artificial boundary (the Piscataqua River) within the New England physiographic region, inconsistent with the known ecology of this region (Foster and Aber, 2004). Further, evidence of precolonial presence of cottontails in northern New England is found in mid-coastal Maine from 500–2500 years ago (Spiess and Lewis, 2001), and writings from the 1600s mention both hares and rabbits (Wood, 1634).

Despite the uncertainties of pre-European landscapes and the associated challenges of identifying historical baselines for wildlife populations, it is likely that New England cottontail abundance and distribution peaked with maximal amounts of shrubland following farm abandonment in the early 1900s (DeGraaf and Yamasaki, 2001; Foster et al., 2002). One example of the abundance of the species at this time comes from a report describing approximately 35,000 rabbits harvested in the state of Vermont in 1944 (Jackson, 1973). The artificially high numbers of early successional species did not persist, as the northeastern landscape changed rapidly again with forest succession, setting in motion the trajectory of early successional habitat loss that has persisted to this day. The historical range of the New England cottontail is typically presented as the range occupied circa 1960, a distribution that was on the downslope of that which peaked with the availability of habitat (Chapman, 1975; Hall and Kelson, 1959; Litvaitis et al., 2006).

**Fig. 3:**
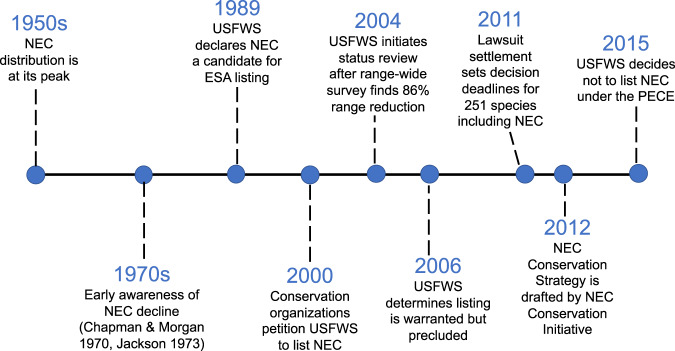
Timeline of events leading to the 2015 USFWS decision not to list the New England cottontail (NEC) under the Endangered Species Act

Awareness of the species’ decline came as early as the 1970s (Chapman and Morgan, 1973; Jackson, 1973), although it was more than a decade before it was formally recognized by USFWS, and another three decades before an ESA determination would be made (see timeline in Fig. 3). By 2004, it was determined that remnant populations of New England cottontails occupied less than 14% of their historical range and less than 10% of the remaining shrubland patches within this range. Subsequent (variably systematic) surveys revealed continued declines, leading to the development of a systematic monitoring program in 2015. Intensive surveys in Maine and New Hampshire (the northern most portions of the species range) revealed a 50% range contraction between 2001 and 2009 (Fenderson et al., 2014), with a census population estimate of <300 individuals in Maine (Litvaitis and Jakubas, 2004) and <100 individuals in New Hampshire (extrapolated from distribution in Fenderson et al. 2014), while the population in Rhode Island, near the core of the species range, was functionally extirpated by 2011 (Brubaker et al., 2014; Fuller and Tur, 2012). Occurrence data used in the 12-month finding (listing decision) were based on these surveys that occurred 2009–2014 and which painted a picture of continued decline (USFWS, 2015a).

**Fig. 4:**
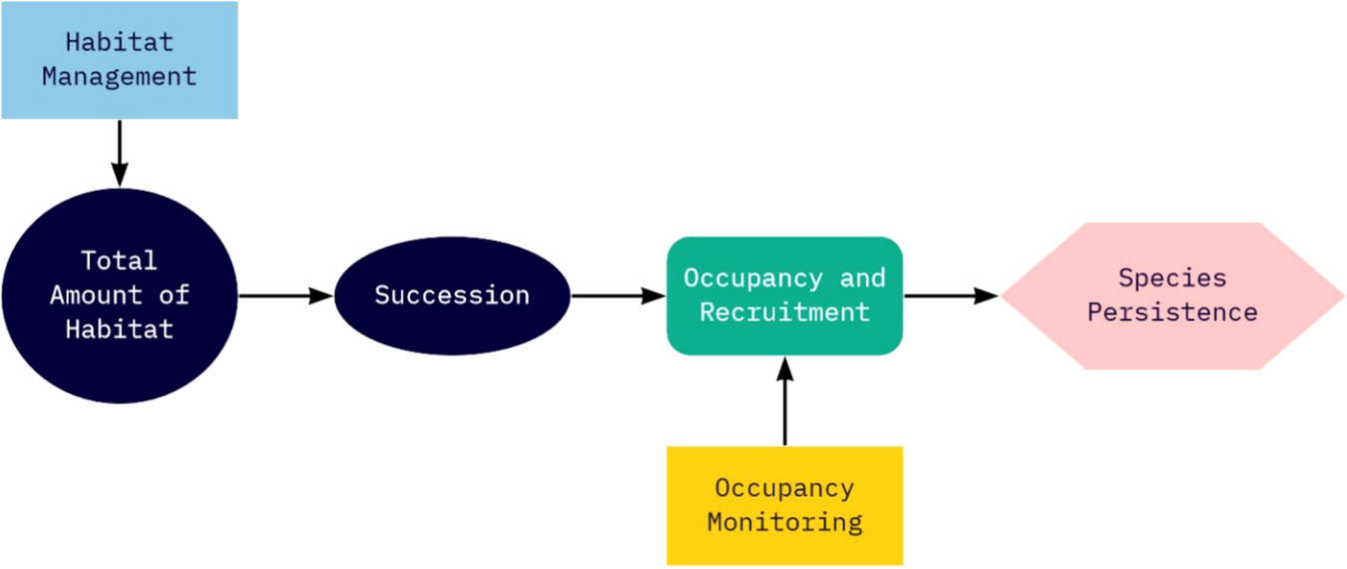
Conceptual diagram for how management action (light blue rectangle) influences population factors (green, round-cornered rectangle) limiting New England cottontail when environmental and ecological processes of total amount of habitat and succession (dark blue circle and ellipse) were perceived as limiting factors to persistence of New England cottontail (pink hexagon) circa the 2012 conservation strategy (Fuller and Tur, 2012). Occupancy monitoring (gold rectangle) provided information to evaluate species response to management actions. The amount of habitat and population size range-wide was not known with certainty at the time of the decision to improve habitat, although evidence pointed toward small populations

Meanwhile, rising awareness about shrubland and early successional forest declines resulted in several initiatives in the Northeast for restoration of these systems – e.g., National Fish and Wildlife Federation Keystone Initiative in Wildlife and Habitat Conservation in which the New England cottontail was one of the focal species. In one of the first implementations of Proactive Conservation in the Northeast, the Range-wide New England Cottontail Conservation Initiative was formed in 2011. This collaborative group of state and federal agencies, nongovernmental organizations, universities, and land trusts developed a formal conservation strategy with measurable objectives for species recovery, including habitat restoration and population goals (Fuller and Tur, 2012). A captive breeding program was initiated, and by 2015, a range-wide occupancy monitoring program was put in place. At the time, knowledge of the species’ ecology was sparse, and limited research and monitoring efforts pointed toward small populations facing isolation, disruption of connectivity, and loss of genetic diversity (Brubaker et al., 2014; Cheeseman et al., 2019b; Fenderson et al., 2011; Fenderson et al., 2014). The decision in 2015 by the USFWS not to list the New England cottontail was based largely upon a PECE analysis that found a high level of certainty that the Conservation Strategy would be implemented and effective. Considerations supporting this decision included the existence of funding mechanisms and commitments of voluntary participation to support an existing Conservation Strategy with explicit measurable objectives (including habitat and population goals), a framework for monitoring, incorporation of principles of adaptive management, evidence of successful management actions since the onset of Cooperative Conservation (e.g., captive breeding program; 3309 ha of habitat managed or planned to be managed in addition to 34,800 ha of existing habitat, exceeding the 14,564 ha needed to implement the Conservation Strategy), and anticipated success of ongoing conservation actions in reaching population goals by 2030 (USFWS, 2015a). The conclusion at the time reflected the assumption that New England cottontail were habitat limited and thereby efforts to create new habitat were sufficient to restore the species (Fig. 4). Since this decision, New England Cottontail conservation has been touted as a success story and a model for the future of proactive conservation for the USFWS. Hereafter, we evaluate the impacts of framing New England cottontail conservation under the PECE and the prospects for successful species recovery, based on the currently available body of scientific knowledge.

## Science Gains Since the PECE Decision

### Population Distribution & Structure

Since the 2015 PECE decision, range-wide occupancy surveys from 2016–2019 suggest a further 50% decline in distribution of New England cottontail from the 2009–2014 surveys (Rittenhouse and Kovach, 2020). Current New England cottontail populations show consequences of recent habitat fragmentation (within the last several decades), evidenced by genetic stochasticity, as populations show signs of genetic drift occurring in isolation (Fenderson et al., 2011). Each of the five geographically and genetically distinct areas identified by Fenderson et al. (2011) have further finer scale population subdivisions, with fragmentation and distances that exceed cottontail dispersal abilities, impeding gene flow among them (Cheeseman et al., 2019b; Fenderson et al., 2014). Each of these distinct subpopulations is characterized by metapopulation dynamics. When sufficiently intact, these metapopulations persist as a network of patches maintained by the processes of colonization and extinction (Hanski and Gilpin, 1991; Litvaitis and Villafuerte, 1996). However, landscape fragmentation has fractured large, single metapopulations into a series of smaller metapopulations; this will likely result in increased rates of decline, as smaller populations face higher extinction rates.

Initial genetic studies in Maine and New Hampshire revealed that metapopulations have low genetic diversity, genetic bottlenecks, and critically low effective population sizes (Amaral et al., 2016; Fenderson et al., 2011; Fenderson et al., 2014). While this knowledge was known at the time of the listing decision, the prevailing notion was that robust populations in the core of the species’ range––Connecticut and eastern New York––persisted as a stronghold. Since that time, a thorough investigation of population genetics, dispersal, survival and home range of New England cottontails in New York strongly contradicts that notion (Cheeseman, 2017; Cheeseman et al., 2019a; Cheeseman et al., 2019b). Cheeseman et al. (2019a) identified nine genetically distinct and isolated subpopulations, with evidence for low genetic diversity and effective population sizes. Similar concerns exist for populations in eastern and western Connecticut (A. Kovach, unpublished data; McGreevy et al., 2021).

In all presently occupied areas, the effective population size (*N*_*e*_) for each metapopulation falls short of the 300 individuals recommended to ensure short term persistence for lagomorphs (Fenderson et al., 2011; Newmark, 1995), and where intensive investigations have been conducted, most identified metapopulations are below the *N*_*e*_ = 50 that is considered the critical lower limit for short-term viability (Bauer, 2018; Cheeseman et al., 2019b; Fenderson et al., 2014). These *N*_*e*_ estimates also point toward consistently low census population sizes across the range. Although empirical population estimates remain limited, recent genetic mark-recapture studies on primarily allopatric populations of New England cottontails in Maine, New Hampshire and Connecticut reveal low numbers and densities typically ranging 0.1-3.75 rabbits per ha, with the majority of patches at <1 rabbit/ha (Kovach and Bauer, 2021; Kristensen and Kovach, 2018). Higher densities on large patches (up to 7 rabbits/ha on patches >5 ha; (Barbour and Litvaitis, 1993) have been reported but have not been observed in recent years. Extending these patch-level estimates to the landscape using knowledge of patch occupancy yields startling low census population sizes in Maine (~150 individuals) and New Hampshire (~50 individuals; Kovach and Bauer, 2021), consistent with estimates from genetically effective population sizes (Fenderson et al., 2011; Fenderson et al., 2014).

While empirical population estimates are lacking for Connecticut, Massachusetts, and New York (the species is known to be functionally extirpated from RI, occurring only on an island breeding colony and two not yet self-sustaining, reintroduction patches), some recent work is informative. In Connecticut, remote sensing efforts have identified 7827 ha of habitat in patches of 2 ha or larger in New England cottontail focus areas (Rittenhouse et al. 2022), and state biologists have used habitat and density to estimate a current population size of 2021 (L. Wahle, Connecticut Department of Energy and Environmental Protection, personal communication). However, the range-wide monitoring survey revealed New England cottontail occupy 0.331 (SE 0.048) of habitat patches surveyed in Connecticut (Rittenhouse and Kovach, 2020). Multiplying the occupancy estimate (0.331) by estimated habitat (7827 ha) indicates only 2591 ha of occupied habitat, which multiplied by 0.77 rabbits per ha of which on average only 33% are New England cottontail (the balance are the sympatric eastern cottontail, *Sylvilagus floridanus*) yields a corresponding population estimate of 668 New England cottontails in Connecticut, a state widely purported as the strong-hold for the species. Similar data on habitat amount and population size are lacking from NY and MA on Cape Cod, yet the few patch-level abundance estimates and the effective population size estimates from genetic data point toward very small metapopulation sizes in these areas as well (Cheeseman et al., 2019b; Kovach and Bauer, 2021; McGreevy et al., 2021); summing across the *N*_*e*_ estimates for the 9 subpopulations in New York leads to a state-wide census population size of 500-1650 (using a *N*_*e*_:census ratio of 3:1 to 10:1). Taken together, this empirical knowledge provides a scenario of critically small effective and census population sizes that are far below those estimated at the time of the 2015 listing decision, and which suggest vulnerability and uncertainty with respect to future population viability.

Remnant New England cottontail metapopulations today persist in small, spatially segregated clusters of patches, restricted to remaining shrublands that are small and discontinuous, within a mosaic of unsuitable matrix. In Maine and New Hampshire, suitable habitat patches are small (2–35 ha, mean = 5 ha) and comprise <5% of the landscape; they are fragmented by roads, development and large blocks of inhospitable landscape (e.g., mature forest or open fields (Amaral et al., 2016). These landscape features strongly influence cottontail dispersal and have likely restricted dispersal relative to historical processes (Amaral et al., 2016). Dispersal of New England cottontails is facilitated by the same shrubby landcover types that comprise occupied patches (Amaral et al., 2016; Fenderson et al., 2014; McGreevy et al., 2021), as well as by linear, anthropogenic features that include shrubby components––roadsides, powerlines and railroads (Amaral et al., 2016). The remaining landscape matrix features—including fields, forests, water bodies, major roads and anthropogenic development—impede dispersal (Amaral et al., 2016; Cheeseman et al., 2019b; Fenderson et al., 2014). The population genetic studies reviewed above suggest that the distribution of critical dispersal habitat is insufficient in the landscape to maintain connectivity among and within populations, resulting in a breakdown of metapopulation function, which jeopardizes future population viability.

### Patch Dynamics

Patch extinction rates are influenced in part by individual fitness, which is in turn influenced largely by habitat quality and climate. In turn, the longer-term persistence of metapopulations relies on the interplay between survival, recruitment, and dispersal (Hanski and Gilpin, 1991). At the time of the listing decision, little was known about New England cottontail patch dynamics, as empirical data on survival, reproduction and dispersal were lacking or limited. Winter survival probabilities for a 60-day period were found to be ~0.35 on small (<3 ha) patches and ~0.7 on large (≥5 ha) patches (Barbour and Litvaitis, 1993; Brown and Litvaitis, 1995), and these researchers suggested that reduced survival and skewed sex ratios were evidence that small patches were functioning as sinks. Since that time, considerable new information has emerged, painting a picture of low patch productivity.

While subject to high variability, average annual New England cottontail survival is ~30% (range 0–75%; (Cheeseman et al., 2021; Kilpatrick and Goodie, 2019, B. Ferry, New Hampshire Fish and Game [NHFG] and A. Kovach unpublished data), suggesting that population persistence relies on high rates of recruitment through births and immigration. However, little is known of New England cottontail reproductive rates and recruitment. Captive individuals produced an average of 2.3 kits per female weaned (2017–2019) with a 45% survival rate to weaning (2015–2019; New England cottontail Regional Initiative performance report 2015, 2016, 2017, 2018, 2019). In a reintroduced population, successful females produced a mean of 2.2 recruited offspring per year (2013–2017), with a maximum of 4; however, not all adult females survived to produce offspring in a given year (Bauer et al., 2020). If these rates are similar in wild populations, this low level of recruitment suggests that, on average, patches have very low or negative population growth and are reliant on immigration for persistence (i.e., population sinks), consistent with declining populations.

Recent radio-telemetry studies demonstrated low rates of effective dispersal that corroborate a mechanism for the population subdivision observed in genetic studies. Cheeseman (2017) documented a 11% dispersal rate for New England cottontails (12 dispersal events out of 108 collared individuals) and did not observe any dispersal events by females. Mean dispersal distance was 911 m; however, only 3 dispersal events greater than 1 km were observed (1.0, 1.5 and 3.9 km). Similarly, a radio-telemetry study in NH identified only a single dispersing male out of 37 collared rabbits (B. Ferry, NHF&G unpublished data). Furthermore, survival studies suggest that dispersal movements are not only high cost, but also that the cost increases with distance such that individuals are unlikely to survive long-distance dispersals (Cheeseman et al., 2021). The genetic and telemetry data together reveal that dispersal cannot overcome fragmentation and habitat patchiness at the landscape level, thereby it cannot effectively offset the low rates of survival and reproduction in the patch. Accordingly, genetic diversity tends to be low and relatedness high within patches (e.g., Bauer (2018)), and these conditions raise concerns about potential for local inbreeding. These findings are underscored by population viability analysis studies of small metapopulations in Maine and New Hampshire, which revealed that population persistence is only likely to be achieved with continuous habitat management, reintroductions, and higher dispersal rates than observed in the wild (Bauer, 2018; Warren, 2017). Collectively, the newly available data on survival, recruitment, and dispersal provide compelling evidence of metapopulation dysfunction in the face of habitat fragmentation.

### Habitat

Habitat quality strongly regulates the fitness and growth of populations and is a driver of both survival and density of New England cottontails (Barbour and Litvaitis, 1993; Brown and Litvaitis, 1995; Cheeseman et al., 2021; Villafuerte et al., 1997). New England cottontails have strict habitat requirements and their home ranges are typically bounded by low-cover patches, potentially because they suffer high predation rates outside dense cover (Cheeseman et al., 2019a; Smith and Litvaitis, 2000). Prior to the listing decision, New England cottontail studies focused on winter habitat use, identifying patches > 5 ha of dense early successional shrublands with >50,000 stems/ha as high-quality habitat. As a result, and due to the finding of low survival in small patches (Villafuerte et al., 1997), prescriptions to create moderate to large dense successional-stage shrub-thickets were favored to promote New England cottontails. More recent studies on space use in relation to patch characteristics have suggested minimum area requirements for New England cottontail persistence may be larger than previously thought, while habitat quality is dramatically influenced by competition, invasive shrubs, and shrubland structure. In patches smaller than 7 ha, cottontails incorporated risky, low-cover habitat into their home range (Cheeseman et al., 2019a), suggesting even moderate sized patches may still be of reduced quality. Further, as small patches that support fewer individuals may be highly susceptible to stochastic extinction, the minimum patch size to maintain a stable population of New England cottontails may be much greater than 5 ha. Simulations of New England cottontail metapopulation viability also revealed the importance of large patches for population persistence (Warren, 2017; Kovach and Bauer, 2021). This finding reinforces the need for large, stable core populations to sustain the more ephemeral peripheral patches in a metapopulation framework.

Moreover, successional systems are particularly vulnerable to invasion by exotic plant species (Johnson et al., 2006), which now dominate shrubland plant communities and alter forest structure and composition in many areas (Silander and Klepeis, 1999). Avoidance of unpalatable invasive shrubs and selection for native vegetation by white-tailed deer (*Odocoileus virginianus*) can exacerbate these invasions where white-tailed deer are overabundant (Elias et al., 2006). This interaction has the capacity to further alter composition of shrublands in favor of invasive shrubs and slow natural succession. While the possibility of invasive shrubs to increase or decrease habitat quality for New England cottontails was recognized prior to the listing decision (Litvaitis et al., 2013), there had been no research on the impact of exotic invasive plants on habitat quality for New England cottontails. Anecdotal observations indicated that where these shrubs were abundant, their removal might result in destruction of critical resources for New England cottontails and patch level extinctions (Litvaitis et al., 2013). Since then, formal study has supported the hypothesis that the architecture of dense invasive shrubs, such as multiflora rose (*Rosa multiflora*) and Japanese barberry (*Berberis thunbergii*), can provide valuable cover resources to New England cottontail and promote survival under certain high-canopy conditions (Cheeseman et al., 2019a; Cheeseman et al., 2018). However, these researchers also noted that invasive shrubs in low canopy early successional shrublands greatly reduce survival of New England cottontails and areas of abundant invasive shrubs may not be selected for when other native dominated shrublands are available.

### Competition

Habitat availability, use, and population density are also negatively impacted by competition with an introduced congeneric, the eastern cottontail. At the time of the listing decision, it was thought that the two species primarily competed for unoccupied patches (Probert and Litvaitis, 1996). Captive studies suggested neither species was competitively dominant, but established territories were typically maintained by the resident cottontail (Probert and Litvaitis, 1996). However, the more generalist habitat requirements of eastern cottontails appear to have facilitated population growth, rapid range expansion, and colonization of common, more open cover types, such as residential areas or the borders of agricultural fields (Probert and Litvaitis, 1996). As a result, they may colonize shrublands at an earlier successional stage, giving them a competitive advantage in successional shrublands within the landscape context (Probert and Litvaitis, 1996).

More recently, research on habitat use by New England cottontails in the presence of competing eastern cottontails suggests that, where eastern cottontails are prevalent, they exclude New England cottontails from otherwise selected early successional patches (Cheeseman et al., 2018). This study found seasonal movement among juxtaposed patches by New England cottontails, which may provide openings into “occupied” territories for eastern cottontail colonization and facilitate displacement of New England cottontails into otherwise not selected late successional shrublands. These authors further noted that eastern cottontail did not select for late successional or ericaceous shrublands, suggesting these shrublands may provide a refuge for New England cottontails. Other work has supported these findings. Occupancy and use by New England cottontails is higher within high-canopy shrublands, while the opposite trend is apparent for eastern cottontails (Buffum et al., 2015; Gottfried Mayer et al., 2018). Additionally, survival of New England cottontails appears to be higher in late successional and persistent shrublands than early successional shrublands that have a large invasive shrub component (Cheeseman et al., 2021). While density of allopatric New England cottontails is highest in early and mid-successional shrublands, in sympatry, New England cottontail density decreases with increasing eastern cottontail abundance in these shrublands; however, eastern cottontails have little to no impact on New England cottontail density in late successional or high canopy persistent shrublands (Cheeseman et al., 2021). These findings have raised concerns that management strategies targeting early successional shrubland conditions to recover New England cottontail may have instead created ecological trap conditions in areas with abundant invasive shrubs or where eastern cottontail are sympatric (Cheeseman et al., 2021). Together, these studies suggest that many management actions undertaken to recover New England cottontail are unlikely to have the desired effect; early successional shrubland creation benefits eastern cottontail but may be harmful or inaccessible to New England cottontail under certain common conditions.

### Disease and Parasites

Until recently, only a single study investigated parasites of New England cottontail. Clancy et al. (1940) found at least one internal parasite species was present in 89% of New England cottontails examined, noting poor body condition in many rabbits infected with 3 or more parasite species. This suggested that multiple infections have a greater impact on rabbit body condition, or perhaps rabbits in poor condition are more susceptible to additional infections. Poor body condition was also reported for rabbits with infections of a single parasite species in the case of intestinal tapeworms or coccidia (Clancy et al., 1940). Considering other lagomorph species, coccidia (*Eimeria* spp.) and nematodes (e.g., *Obeliscoides cuniculi*) are commonly reported and have been associated with morbidity, mortality, or reduced survival in these hosts (Duszynski and Couch, 2013; Lello et al., 2005; Murray et al., 1997). While the vast research on lagomorph parasites and the effects of parasites on individual survival records of parasite induced population decline were recognized, the work of Clancy et al. (1940) was not considered and parasites and disease were not deemed a threat in the 2015 finding on the status of New England cottontail (USFWS, 2015a).

More recently, parasites and their potential impacts have been investigated (Mello, 2018; Whipps et al., 2019) with an emphasis on surveying sympatric populations of New England and eastern cottontails. The concern is that nearly panmictic eastern cottontails may act as reservoirs for pathogens that can also infect patchily distributed New England cottontails. Reservoirs can provide a potential source of infection or maintain a pathogen in an ecosystem at levels that would not be observed in the threatened species alone, thus increasing the likelihood of a negative impact on the threatened host (De Castro and Bolker, 2005). In a study on gastrointestinal parasites, Whipps et al. (2019) found two nematode species and numerous coccidia in both cottontail species. These same types of parasites have been implicated in negative outcomes for rabbits. Virulent bacterial and viral pathogens are not well characterized in New England cottontail but are recognized problems in other rabbit species and may pose future threats to the species.

Concerns over ectoparasites, particularly ticks, have also arisen. High tick burdens, in line with burdens implicated in mortality and population crashes of eastern cottontails, have been observed in New England cottontails (Mello, 2018; Smith and Cheatum, 1944). Indeed, high tick burdens were implicated as a cause of mortality in their study. More recently, tick burdens have been associated with drastically reduced survival of juveniles under certain conditions and may be a contributing factor to adult mortality (Cheeseman et al., 2021). While historical surveillance of disease and parasites are lacking, these recent findings suggest they pose additional current threats to New England cottontail. In their review of the literature on the impact of infectious diseases on species loss, Smith et al. (2006) attributed disease to approximately 4% and 8% of cases of extinction and endangerment, respectively. Included in the many challenges of understanding these impacts on any species are a lack of monitoring, incomplete historical data, and the variable impacts of different diseases.

## Remaining Uncertainties & Threats

**Fig. 5:**
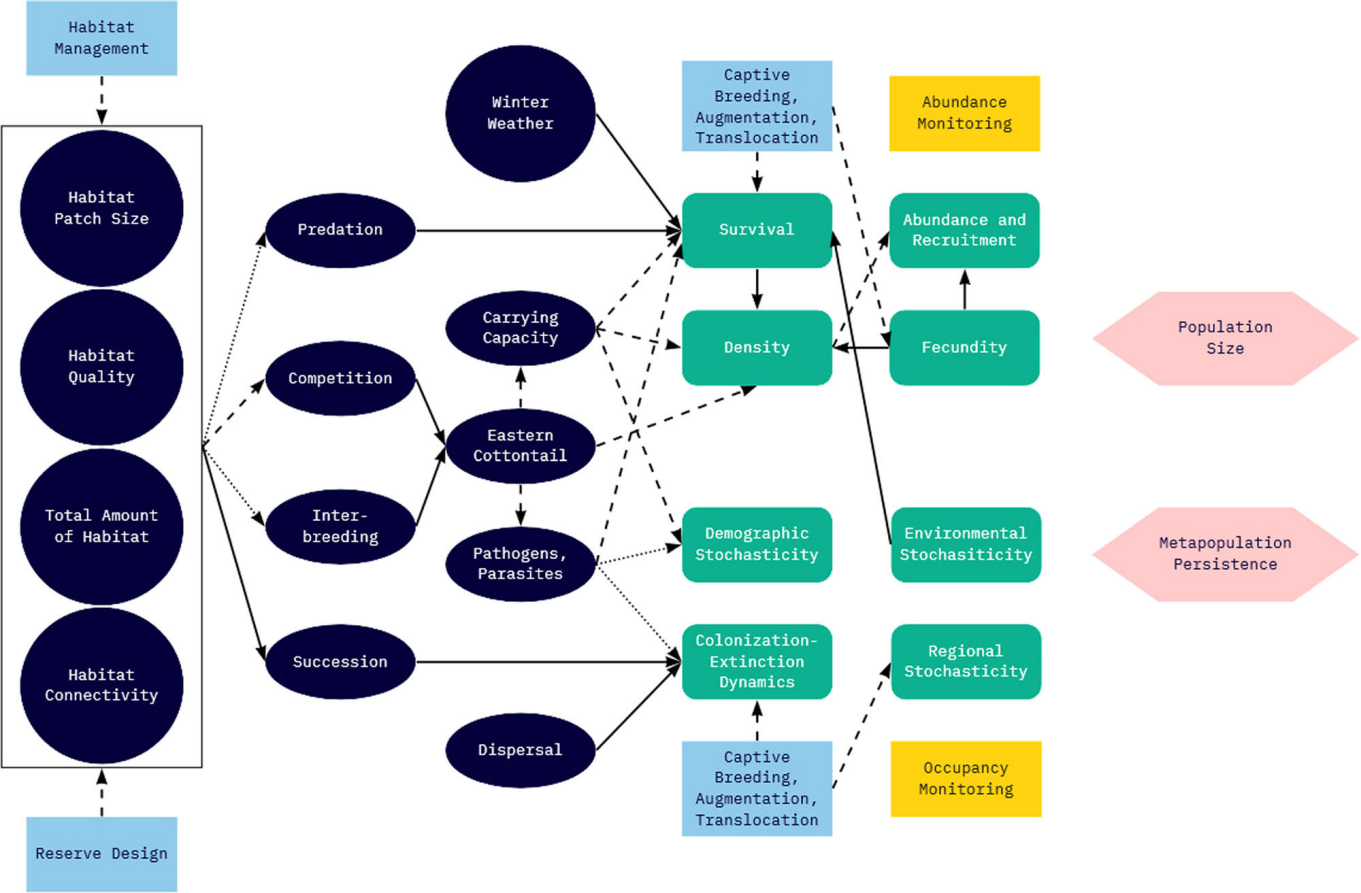
Conceptual diagram for how management actions (light blue rectangles) and environmental and ecological processes (dark blue circles and ellipses) influence population and metapopulation factors (green, round-cornered rectangles) limiting New England cottontail population size and metapopulation persistence (pink hexagons). Abundance and occupancy monitoring (gold rectangles) provide information to evaluate species response to management actions at population and metapopulation levels (pink hexagons). Scientific studies since the PECE decision have confirmed some relationships (solid lines) and reduced some uncertainty (dashed lines) in the relationships between habitat patch size and New England cottontail population size, response to habitat management actions, and contributions of reintroduction from captive breeding to population size, though substantial uncertainty exists for other relationships (dotted lines). Complex relationships among population factors and population size, and among metapopulation factors and metapopulation persistence omitted for diagram clarity

Scientific studies since the PECE decision have provided substantial information about New England cottontail ecology, management, and conservation, and now provide a more robust picture of the management decisions under consideration (Fig. 5). Despite this new information, key assumptions and uncertainties remain, and new threats to New England cottontail have emerged.

### Assessing trends

The regional monitoring program, initially developed and implemented in 2015, was designed to assess occupancy, changes in occupancy over time, and response to habitat management by New England cottontail (Shea et al., 2019). After two years of implementation, the regional monitoring effort was shown to be ineffective in tracking occupancy trends due to low power to detect a large change in occupancy between years (Rittenhouse and Kovach, 2020). The initial survey protocol, established with a random sampling approach, failed to account for the extremely low occupancy rate of New England cottontails in suitable patches. The protocol was revised in 2017 to focus on surveying patches known to be occupied or in dispersal distance of patches known to be occupied; subsequently it yielded high power to document trends, and also revealed that a 50% decline in occupancy has occurred over the last decade (Rittenhouse and Kovach, 2020). With the improved survey design, the effort now provides information regarding occupancy, trends in occupancy, and responses to habitat management actions (Rittenhouse and Kovach, 2020).

Across all years of the regional monitoring effort (2016-2017 through 2020-2021 surveys), New England cottontail occupied 224 of 663 sites sampled and occurred exclusively (without eastern cottontail) on only 75 of those 224 sites (Table 1). When considered in context of the resiliency, representation, and redundancy tenets of species status assessments (USFWS, 2016), the New England cottontail does not appear to be resilient to current habitat and landscape conditions, extreme winter weather (Bauer et al., 2020; Cheeseman et al., 2021) nor to sympatry with eastern cottontail, as evidenced by the increasing proportion of co-occurrence with time (Table 1). Representation is low given the species’ narrow, specialized niche and concerns for low genetic diversity. Redundancy is also low and declining with only 224 sites (patches) in the remnant distribution occupied over the past 5 years (Table 1). Considering the relatively small number of known occupied patches range-wide (Table 1), alongside the relatively low patch-level densities, the range-wide population can be expected to be as low as 3000 individuals or less, range-wide. This is a startling low number for a lagomorph and substantially lower than the 17,000 individuals extrapolated from available shrubland, used as a population estimate in the 2015 listing decision (USFWS, 2015a). Further empirical evaluation of this range-wide population size is a key remaining uncertainty in the ongoing conservation effort. While population size is a difficult parameter to estimate for cottontails, given cryptic behaviors and low trap rates, it can be estimated by incorporating recently developed abundance estimation approaches into the existing monitoring program (Kristensen and Kovach, 2018). Current uncertainties in these estimates notwithstanding, the critically low abundances observed to date, in conjunction with the observed breakdown in metapopulation function (low survival, reproduction and dispersal; see above) pose imminent threats to population persistence.

**Table 1:** New England cottontail occurrence and co-occurrence with eastern cottontail from the regional monitoring effort spanning Connecticut, Massachusetts, Maine, New Hampshire, New York, and Rhode Island, USA, for the survey years 2016–2017 through 2020–2021 (Rittenhouse unpublished data [regional monitoring report for 2020–2021; New England Cottontail Regional Initiative 2021])

Survey year	Sites sampled	Sites with New England cottontail	Sites with eastern cottontail	Sites with co-occurrence	Sites with NEC only	Proportion co-occurrence
2016-2017	213	46	107	27	19	0.127
2017-2018	338	103	179	58	45	0.172
2018-2019	378	135	187	78	57	0.206
2019-2020	220	99	111	58	41	0.264
2020-2021	167	93	125	55	38	0.329
Total (all years)	663	224	376	149	75	0.225

### Response to Management

Widespread loss of shrublands has resulted in a heavy reliance on anthropogenically created successional shrublands by New England cottontails and a propensity for wildlife managers to create these shrublands to promote New England cottontails (Fuller and Tur, 2012). Yet, New England cottontail populations have failed to respond to these management efforts. Among managed patches, few support or provide suitable habitat for New England cottontail at present (Litvaitis et al., 2021; Rittenhouse and Kovach, 2020). Many managed patches are too young, too small, too far from existing populations to support sustainable New England cottontail populations or sites simply do not possess the characteristics (e.g., soil, disturbance/management history) necessary to support shrubland development, and thus provide habitat (Litvaitis et al., 2021). Occupancy remains higher in unmanaged patches than in patches managed for New England cottontail in three out of four years with data (Rittenhouse unpublished data [regional monitoring report for 2019-2020]). Colonization by New England cottontail has been observed in a minority of patches, consistent with dispersal limitations (Amaral et al., 2016; Cheeseman, 2017; Cheeseman et al., 2019b; Fenderson et al., 2011; Fenderson et al., 2014), while the number of sites occupied by New England cottontail have continued to decline (Rittenhouse and Kovach, 2020). New England cottontail density is not associated with patch management status in Maine and New Hampshire (Kovach and Bauer, 2021), nor is occupancy, range-wide (Bischoff, Rittenhouse and Rittenhouse, unpublished). Results from recent simulation modeling support this notion. Kovach and Bauer (2021) found that a small, remnant population simulated with a 9-fold increase in habitat through restoration failed to achieve the conservation target size of 500 after 100 years. Only through habitat restoration combined with multiple population augmentations over time and optimistic vital rates (values representing the best-case scenario from the literature) was this simulated population able to increase to the 500-individual goal and persist. Simply put, the last 10 years are evidence that we do not have enough information to confidently suggest we can restore habitat to recover New England cottontails.

### Captive Breeding and Reintroductions

A captive breeding program was established in 2011 with the goal of supplying 500 rabbits for reintroduction per year (New England Cottontail Regional Initiative, 2014). These efforts now include two zoos, two outdoor pens and two island breeding colonies (New England Cottontail Regional Initiative, 2019). While these efforts have successfully produced 382 kits from 2011-2021 (including 2 strong years producing 96 in 2018 and 70 in 2019, followed by the COVID-19 pandemic limited breeding and releases in 2020 and 2021), initial production goals have yet to be realized (New England Cottontail Regional Initiative, 2017, 2018, 2019). These successes have been limited in part due to a combination of logistical constraints, limited zoo and pen resources/capacity, and uncertainties regarding sustainable take from island populations, as well as knowledge gaps regarding small litter size and low juvenile survival to weaning that may limit these efforts.

Captively-bred New England cottontails have been reintroduced in groups of 7-21 individuals across 2-5 years at five sites – Great Swamp and Ninigret NWR in RI, Bellamy Wildlife Management Area and Rollinsford in NH, and Wells Reserve in Maine. Reproduction in the wild (of both founders and their wild-born offspring) has been observed in at least three reintroduction sites, however, post-release and annual survival is highly variable, as is reproductive contribution among founding individuals. Achieving a self-sustaining population requires persistent effort, and without annual augmentation via new releases, some monitored populations declined to low numbers after an initial period of growth, with biased sex ratio and a loss of genetic diversity (Bauer et al. 2020, Bauer and Kovach unpublished data). In two recent releases, dispersal into the surrounding landscape has occurred at the time of the writing of this paper, potentially indicating a more robust landscape-level response (Bauer & Kovach, unpublished data). These findings underscore the challenges of bringing back an extirpated population in a landscape devoid of neighboring occupied patches; such connectivity is critical in a metapopulation context to provide dispersers and gene flow to offset winter mortality and bolster genetic diversity (Bauer et al., 2020). The small numbers of individuals released and the small habitat patches available for reintroduction provide further challenges for these efforts. Other successful lagomorph reintroductions have released orders of magnitude larger numbers of individuals, because high mortality and low rates of successful establishment are common (DeMay et al., 2017; Watland et al., 2007).

### Limits to Population Growth

Integrating the most current knowledge of status, threats and remaining uncertainties, we have identified a number of factors that appear to be limiting growth and recovery of New England cottontail populations. First, stochastic factors – demographic, genetic and environmental––are at play in the remnant, small populations. Random demographic fluctuations result in skewed sex ratios (Bauer et al., 2020), and winter storms, especially late spring snows, lead to stochastic mortality events (Bauer et al., 2020; Cheeseman et al., 2021). Genetic bottlenecks have reduced genetic diversity and continued ongoing genetic drift in isolated populations may result in loss of important adaptive alleles (Cheeseman et al., 2019b; Fenderson et al., 2011; Fenderson et al., 2014). High relatedness of individuals on small patches, with limited dispersal, suggests inbreeding may be ongoing, although the latter has not been confirmed with fine-scale genetic data. Low heterozygosity and inbreeding may limit population growth through reduced fitness, survival and reproduction (Saccheri et al., 1998).

Low vital rates are another primary factor limiting population growth. Additionally, mortality rates may be elevated above evolutionary baselines due to habitat degradation (i.e., invasive plants, small patch size), increased tick burdens with climate change, and the naturalization of novel predators like coyote and recovery of native predators like bobcat and fisher (Broman et al., 2014; Cheeseman et al., 2021; Gompper, 2002; Hapeman et al., 2011; Ogden et al., 2021). Further, density dependent effects that release small populations from predation pressure by generalist predators (e.g., predator population regulation, prey switching) may not operate in favor of New England cottontail where the similar but more abundant eastern cottontail is present. In such cases, it is possible for predation to drive the species with a lower survival capacity to extinction (Korobeinikov and Wake, 1999). Further, population expansion, including into managed patches, is limited by low colonization rates––a result of low vagility, low habitat availability, and a highly fragmented matrix. This is particularly concerning as many shrublands are ephemeral and, thus, suitable patches are continuously lost to natural forest succession. Without colonization of new patches to balance ongoing patch extinctions, populations will continue to decline.

Although thought to be rare, hybridization with eastern cottontail has recently been documented (New England cottontail Regional Initiative, 2018, 2021). These recent observations may indicate rates of hybridization are increasing in areas where New England cottontail is on the decline and in low numbers on a patch and where eastern cottontails are newly colonizing an area previously occupied by New England cottontails only. Wasted reproductive effort from such inter-specific mating (failed or successful) further impedes a positive population trajectory on local patches, and recent evidence that F1 hybrids can reproduce (New England Cottontail Regional Initiative, 2018, 2021) raise additional questions about potential threats of genetic introgression.

Acute, and potentially severe infections with bacterial and viral pathogens are also of concern for cottontail rabbits. Notable examples are *Francisella tularensis* (cause of tularemia) and the rabbit hemorrhagic disease virus serotype 2 (RHDV2). The bacterium *F. tularensis* is highly infectious and zoonotic, with rabbits considered a natural reservoir and amplifying host (Brown et al., 2015; Hestvik et al., 2015; Petersen and Schriefer, 2005; Wobeser et al., 2009). Virulence varies by bacterial strain, but the more virulent strains, which are common in the United States, can cause mortality within 1 week of exposure (Brown et al., 2015). In a study of a wild population of eastern cottontails in Illinois, where tularemia was enzootic, Woolf et al. (1993) reported almost a third of collared animals succumbed to this disease during the study period, illustrating the potentially devastating effects such infections can have. Arthropods are known vectors of *F. tularensis*, and particularly ticks (Brown et al., 2015; Hestvik et al., 2015), which is relevant for New England cottontails given the tick burdens mentioned above. Studies on tularemia specifically in New England cottontails are lacking, but a recent news article reports a case on Patience Island, Rhode Island (News, 2021).

The most recent threat, RHDV2, may be the most serious one to the future persistence of remnant New England cottontails. Rabbit hemorrhagic disease virus is well documented in European rabbits and has a high fatality rate at 70-100% (Dalton et al., 2012; Kerr and Donnelly, 2013). Cases in the United States have been reported sporadically (Kerr and Donnelly, 2013; McIntosh et al., 2007) but a recent outbreak in wild rabbits in the Southwestern US has sparked concerns of more extensive spread (Asin et al., 2021). Biosecurity and minimizing the spread of this virus has been emphasized (USGS, 2020a, b, c) because once established, the disease can be devastating. Reports on the susceptibility of *Sylvilagus* species to RHDV2 vary. The recent cases in the United States include several cottontails including the eastern cottontail, desert cottontail (*Sylvilagus audubonii*), and mountain cottontail (*Sylvilagus nuttallii*) (Asin et al., 2021; Lankton et al., 2021). Because of its overlapping range with the New England cottontail, the eastern cottontail could be an important reservoir of infection if RDHV2 were to spread to the Northeast. These findings suggest that the potential risk to New England cottontail could be high.

In summary, in 2015, the 12-month finding identified habitat loss as the most significant threat to New England cottontail, with predation, competition with eastern cottontails and small population size noted as contributing factors. In our review, we have emphasized numerous additional factors, primarily biological (low vital rates, connectivity, genetic variation, hybridization, parasites and disease), that contribute to the continued decline of the species. Thus, it is now evident that habitat restoration alone is insufficient to reverse the population decline of the New England cottontail, at least in the near term.

A more successful path toward recovery will require long-term commitments and a multi-scale approach to management, where we not only consider habitat within the patch, but the arrangement of patches on the landscape and intervening matrix, with landscape-level planning that considers succession dynamics in this system. It will also require dedicated resources to facilitate an enhanced captive breeding effort that produces and releases hundreds of rabbits annually on the landscape, until populations stabilize and become self-sustaining. Existing simulation models (Bauer 2018; Kovach and Bauer, 2021) can be used to predict numbers and rates of releases required for successful reintroductions, as well as for managing removals from captive breeding colonies. Once these reintroductions and augmentations stabilize, it is conceivable that further captive breeding may not be needed, if habitat management can maintain these rebuilt populations. Successful recovery will also require an improved understanding of and mitigation of threats, as they evolve, and the incorporation of new knowledge into management, as it becomes available. Doing so will require dedicated funding for research and adaptive management experimentation, and potentially bringing in guidance from experts outside of the current initiative, to move beyond ad hoc approaches. Continued, consistent support for the monitoring program is also critical for tracking trends and understanding their causes; expanding monitoring to assess abundance in addition to occupancy will be important to accurately evaluate population status. Recent declines in financial support of the monitoring program and future uncertainties about funding priorities in the face of the shift away from a dedicated New England Cottontail Conservation Initiative toward a broader Young Forest Initiative must be addressed. In the big picture, open recognition of the conservation reliant nature of the New England cottontail, and accordingly, a long-term strategy for its conservation, with consistent resource commitments, is necessary.

## Synthesis and Assessment of the Conservation Initiative

The New England Cottontail Conservation Initiative brought substantial efforts and successes from the cooperative effort of all partners involved. One important success of this cooperative effort has been its motivation of cross-jurisdiction sharing of information and collaborative research that has now reduced several of the aforementioned sources of uncertainty (and revealed new ones), with ongoing learning gains. The Conservation Initiative comprises dedicated and motivated partners in science, management, and conservation, who have helped facilitate studies and implement restoration actions. Leveraging resources across partners and organizations has facilitated progress without ESA support, albeit perhaps slower and with more difficulty than might have been. The burden of financial support and conservation effort was placed on the cooperating states and other partners, which focused on implementing the Conservation Strategy with limited resources and infrastructure and a mismatch of recovery goals with species status and threats.

Our review of the species status and ecology shows New England cottontail recovery is far from achieved and calls into question the 2015 decision declining ESA support and protections. Reliance on the PECE policy for the New England cottontail ESA listing decision was predicated on the assumption that habitat was the primary factor limiting populations and consequent optimism that habitat management and time would lead to recovery, despite substantial uncertainty in: 1) estimates of abundance and tools for tracking trends, 2) effect of invasive eastern cottontails and resulting niche partitioning on sympatric patches, 3) factors limiting population growth, especially dispersal ability, survival and reproductive rates, 4) best practices for creating high quality habitat in a useful time frame, and 5) the success rate for augmenting or founding wild populations from captive-bred animals. Consequently, the conservation actions have been less effective than anticipated and the risk of extinction has increased, rather than decreased, in the subsequent seven years. New England cottontail conservation thus demonstrates the pitfalls of substituting cooperation for species status assessment, which is intended to be the overriding basis for listing decisions (Uchitel, 2005).

A major consequence of the Proactive Conservation paradigm has been an inadequacy in the metrics used to track success of conservation actions with respect to species status. For species listed under the ESA, metrics of success are typically measured in the form of species status, population trends, and PVAs (Evans et al., 2016). Under the Proactive Conservation model, unlike when a species is listed, the metric of success is not clearly defined. Collaborative groups often focus on impacts that are intermediate from a conservation standpoint, e.g., conservation easements, agreements, meetings, and establishment of committees, and rarely measure environmental outcomes, such as species or population response (Reid et al., 2010). Similarly, for New England cottontail conservation to date, the measure of success has largely been expressed as numbers of acres of habitat managed, restored or created, without evidence that habitat acreage translates into rabbits. Tracking the response to management in terms of population numbers, while critically important, has been largely absent from the conservation effort (New England Cottontail Regional Initiative, 2021). This focus on habitat management has resulted in the majority of funding through the Conservation Initiative being relegated to habitat projects, with a modest, unstable amount for monitoring (occupancy and translocations) and minimal dedicated funding for research. As a result, tracking population trends has been slow, monitoring abundance virtually not possible except via separately funded initiatives of academic researchers, and research has been supported either by state funding or through other, very limited applied research funding. Thus, the uncovering of additional threats and filling gaps in key ecological knowledge has been slower than it might have been, and why so many uncertainties remain today (section III). Additionally, the lack of focus on population numbers has resulted in disparate perceptions and misconceptions among stakeholders about the status of the species.

How might have ESA listing improved the conservation outcomes for the New England cottontail? While we recognize that listing the New England cottontail under the ESA would not have quickly removed the threats we have revealed here, we believe that it would have led to differences in the process and outcomes. Firstly, listing the New England cottontail as endangered under the ESA would have resulted in a clear understanding among stakeholders and the public about the dire status of the species and accordingly, the imminent need for rapid and large-scale interventions. Secondly, it would have framed the recovery effort and goals in terms of quantitative metrics of population sizes and viability projections, rather than acreage of habitat on the landscape. Accordingly, the initial monitoring program would have been designed as for a rare species, targeting remnant populations (as with the revised protocol) rather than broader surveys of available habitat more appropriate for a species that is widespread or has an increasing trajectory. Knowledge gaps with respect to population status would have been filled by targeted abundance monitoring and population modeling, which has only recently been considered under the cooperative effort, as the status of the species has become more apparent. Thirdly, the periodic review of a recovery plan that is required under the ESA would have more readily identified trends and met them with corresponding action. Ultimately, we believe that, with ESA listing, evaluations of status, threats, uncertainties, and response to management would have come sooner, due to targeted knowledge-seeking and recovery-tracking efforts. Section 6 funding would have supported the recovery effort and may have resulted in dedicated commitments and support for a larger, more extensive captive breeding effort. A recovery team would have governed range-wide decision-making and prioritization of resources and actions among stakeholders, which is currently based largely on equitability. Listing would have made recovery a mandate for all federal agencies, ensuring long-term support for all of the partners engaged in conservation, in contrast to the state of uncertainty that exists today for the future support of the initiative. These highlighted differences in the process and outcomes of the conservation effort relative to that under ESA protection are likely generalizable to scenarios for other at-risk species for which Proactive Conservation has averted (or will avert) ESA listing.

## Evaluating the Use of PECE in the Proactive Conservation Paradigm

In the bigger picture of at-risk species conservation, a successful path to recovery warrants evaluation of the limits of Proactive Conservation, in particular, the application of the PECE to preclude species listings. Listing under the ESA may be viewed by regulatory agencies and stakeholders as a hindrance to conservation because of the penalties, permitting requirements and other legal burdens imposed by the ESA (Wyman (2008)). Moreover, listing may be viewed by some as a conservation failure in and of itself, because so few species have been delisted due to recovery, which under ESA definition is reached when a species no longer needs the protections of the Act (Adler, 2017; Stokstad, 2005). Although Proactive Conservation, in conjunction with PECE, fulfills a desire to find mechanisms other than the ESA to implement species conservation, our case study highlights that this paradigm may have unintended consequences and limitations with respect to facilitating species recovery.

The distinct roles for science and policy are recognized as necessary for transparency and consistency in species assessments and decision-making under the ESA (Smith et al., 2018; Waples et al., 2013). Yet, in the New England cottontail PECE decision, the lines between scientific data collection and policy judgement were not distinct. The ESA mandates that listing decisions be based only on the “best scientific and commercial data available” (ESA sec. 4(b)(1)(A)). Failure to follow this mandate has become a recurring critique in recent listing decisions (Lowell and Kelly, 2016; Murphy and Weiland, 2016; Evans et al., 2016). In response to this critique and additional concerns about the intrusion of politics into listing decisions, the USFWS has adopted a revised assessment process, the Species Status Assessment (SSA), which incorporates scientific advancements since the ESA was first developed (Smith et al., 2018; USFWS, 2016). The SSA, in use for ESA decisions since 2017, prescribes a three-step approach that, via thorough review of existing literature and, if needed, new expert analyses, documents the ecology, current condition and future condition of a species. The result is a scientific report that characterizes extinction risk. The process involves not just listing of threats, but also a thorough evaluation of their risks; accordingly, it relies heavily on quantitative analyses, including modeling and simulations, and explicit consideration of uncertainty with respect to future condition. This robust process provides a defensible framework for science-based decision making. However, under the current Proactive Conservation paradigm, the outcome of this assessment with respect to a listing decision can be over-ridden by the outcome of a PECE evaluation.

The PECE allows Proactive Conservation to be considered in listing determinations. It does not, however, allow decision makers to ignore the best available scientific and commercial data, and prohibits consideration of collaborations newly assembled with the intent of avoiding listing (USFWS, 2003b). The use of the PECE to support not listing the New England cottontail asserted certainty in the outcome of management, and at the same time stated that management would be done adaptively to reduce uncertainty (USFWS, 2015a), which would appear to recognize that knowledge at the time was inadequate. It may be that, at the time, adaptive management was viewed as something to be employed for minor adjustments in best practices. Adaptive management has, in fact, grown out of the Cooperative Conservation process for New England cottontails, and while this success is attributable to the collaborative nature of the effort, it has highlighted major deficiencies in knowledge of the species ecology that require major rather than minor adjustments in practices. Yet, due to the PECE decision and declaration of successful recovery, policy makers now have an understandable misperception that the species is secure, despite the findings to the contrary that we have detailed in this paper, and the conservation team is facing the threat of diminishing funds. This highlights the problem with using the PECE to preclude listing in the face of either insufficient knowledge or uncertainty about species status. While proactive conservation efforts are valuable in motivating diverse networks of stakeholders to take positive management action, they are an inappropriate substitute for species status assessments and become problematic when implemented as a means for avoiding listing rather than achieving species recovery. Indeed, this is illustrated by several recent and controversial listing decisions, including greater sage grouse, lesser prairie chicken (*Tympanuchus pallidicinctus*), and dunes sagebrush lizard (*Sceloporus arenicolus*), for which the PECE decision took into account variable voluntary actions, despite uncertainty in their effectiveness and evidence for highly vulnerable species status. In all three cases, new proposals for ESA protection are currently under review (BLM 2021, USFWS 2020, 2021).

Listing a species does not preclude Cooperative Conservation. In fact, the recovery imperatives of the ESA often compel collaborative efforts, and the Section 10 provisions dealing with incidental take permits facilitate agreements between federal and non-federal entities (Fischman et al., 2021). The federally threatened piping plover (*Charadrius melodus*) is an example of a species facing numerous controversies related to conflict between shoreline use and conservation, yet under the ESA a large international coalition of federal, state, municipal, and private partners has worked for over three decades to bring the species to where it is likely secure from stochastic extinction (Hecht and Melvin, 2009). Yet the piping plover has not been heralded nationally as a success the way the New England cottontail was, likely because it remains on the ESA list. In recognition of the conservation-dependent status of the piping plover, one of the species recovery goals requires the establishment of long-term agreements by landowners to continue conservation measures after de-listing. Based on all the information we have compiled on the New England cottontail since its listing decision, it is also a conservation-dependent species.

## Conclusion

The decision to not list the New England Cottontail under the U.S. Endangered Species Act was heralded as one of the greatest wildlife conservation success stories of its decade, with the success attributed specifically to Proactive Conservation. Yet, the published evidence at the time pointed only to a species that was in trouble, and seven years of collaborative effort have not prevented further declines. The listing decision, with application of the PECE, illustrates the role of factors other than scientific data in making determinations of species status. The need for a reckoning between scientific and nonscientific factors that affect listing decisions has long been recognized (Doremus, 1997), with potential implications for amendments to the law or new approaches to its implementation.

We see two resolutions that may help facilitate listing of at-risk species like the New England cottontail, where the scientific data strongly support protection, but regulatory agencies see listing as a possible barrier to conservation. The first is to remove the stigma of failure from listing. Although it is true that few species have been removed from the list because they can persist without protection, very few listed species have gone extinct (Smith et al., 2018). The ESA therefore clearly fulfills its primary goal. To reduce the stigma of the listing decision, Quarles (2010) recommended redefining recovery under the ESA but did not provide a new definition. We believe that recovery should not depend on delisting, in recognition that few species will ever be delisted because they require intervention to persist alongside human activity. As an alternative, we suggest recovery be redefined based on security from extinction under existing conservation measures, and that ESA regulations could be relaxed, such as through Section 10 measures, when this security threshold is reached. Such measures have been used in the case of the piping plover in recent years. Second, we recommend the continued use of 4(d) to tailor specific conservation measures for species listed as threatened (Fischman et al., 2021). We believe that the majority of “warranted but precluded” species, if listed, would be designated as threatened rather than endangered, and that 4(d) rules would promote the continuance of cooperative conservation that may have been initiated under candidate agreements, while not necessarily bringing the punitive measures of the ESA to bear.

Lastly, we advocate strongly for the use of the SSA process in listing decisions, and for the status of a species, based on the SSA, to be clearly articulated prior to a PECE analysis. If a SSA determines that a species is at risk of extinction and warranting protection, this information should be presented clearly to all stakeholders, regardless of ongoing conservation efforts. Application of the PECE analysis should require greater transparency around uncertainty surrounding conservation outcomes. Achieving certainty in the success of conservation efforts is a tall order to demonstrate and it requires evidence; often we know very little about how to implement successful restoration or how impactful our efforts will be, and we need to acknowledge and account for these limitations. This point is relevant and timely, given current at-risk species that are on the docket for upcoming listing decisions, especially those facing threats of climate change and sea-level rise, such as the saltmarsh sparrow (*Ammospiza caudacuta*), for which very little evidence exists to evaluate the likelihood of success of recently implemented restoration activities.

Extinction of a species represents the failure of the ESA, no matter how cooperative the conservation effort and no matter how much was learned in the process. Extinction of a species that was known to be imperiled but was never listed is the worst possible outcome, as it represents the failure to uphold a public trust. If the status quo continues, the New England cottontail will continue to face a high risk of that outcome (Litvaitis and Lanier, 2019), despite the many, notable efforts of extremely dedicated conservation partners in the New England Cottontail Conservation Initiative, who are committed to truly adaptive efforts to recover the species. Without regulatory support of the ESA, the initiative is in danger of losing its funding, due to the misperception that the problem has been solved, and the initiative’s efforts to establish the infrastructure, decision-making framework, and periodic review to meet conservation targets have yet to trigger a review of the process and ensure the success of its collaborative efforts.
